# Correspondence between practitioners’ self-assessment and independent motivational interviewing treatment integrity ratings

**DOI:** 10.3389/fpsyg.2022.890579

**Published:** 2022-07-26

**Authors:** Maria Beckman, Helena Lindqvist, Lina Öhman, Lars Forsberg, Tobias Lundgren, Ata Ghaderi

**Affiliations:** ^1^Centre for Psychiatry Research, Department of Clinical Neuroscience, Karolinska Institutet, and Stockholm Health Care Services, Stockholm, Sweden; ^2^MIC Lab AB, Stockholm, Sweden; ^3^Division of Psychology, Department of Clinical Neuroscience, Karolinska Institutet, Stockholm, Sweden; ^4^Stockholm Centre for Eating Disorders, Stockholm County Council, Stockholm, Sweden

**Keywords:** Motivational interviewing (MI), treatment fidelity, evidence-based treatments, MITI, self-assessment, metacognition

## Abstract

As evaluation of practitioners’ competence is largely based on self-report, accuracy in practitioners’ self-assessment is essential for ensuring high quality treatment-delivery. The aim of this study was to assess the relationship between independent observers’ ratings and practitioners’ self-reported treatment integrity ratings of Motivational interviewing (MI). Practitioners (*N* = 134) were randomized to two types of supervision [i.e., regular institutional group supervision, or individual telephone supervision based on the MI Treatment Integrity (MITI) code]. The mean age was 43.2 years (SD = 10.2), and 62.7 percent were females. All sessions were recorded and evaluated with the MITI, and the MI skills were self-assessed with a questionnaire over a period of 12 months. The associations between self-reported and objectively assessed MI skills were overall weak, but increased slightly from baseline to the 12-months assessment. However, the self-ratings from the group that received monthly objective feedback were not more accurate than those participating in regular group supervision. These results expand findings from previous studies and have important implications for assessment of practitioners’ treatment fidelity: Practitioners may learn to improve the accuracy of self-assessment of competence, but to ensure that patients receive intended care, adherence and competence should be assessed objectively.

## Introduction

Treatment fidelity is defined as the extent to which a treatment is delivered according to a given standard (Hogue et al., 2015). It includes two factors: (1) *Adherence* (i.e., the extent of proposed treatment components present in the session); and (2) *Competence* (i.e., practitioners’ skills) (Beidas et al., 2014b; Bearman et al., 2017). Assessment of treatment fidelity is a crucial part of understanding if and how a treatment works, and thus an important part of the development of all evidence-based treatments (EBT) (McLeod et al., 2013). Fidelity assessment is also key for the dissemination and implementation of EBT, as it is a prerequisite for both effective training of practitioners and quality assurance of clinical practice (McHugh et al., 2009; Hogue et al., 2015). Primary methods of measuring fidelity are direct (i.e., monitoring of sessions) or indirect (i.e., practitioners’ self-report) (Beidas et al., 2014a). However, results from a number of studies highlight the inaccuracy of practitioners’ self-report (Carroll et al., 1998, 2010; Brosan et al., 2008; Beidas and Kendall, 2010). These weak correlations between practitioners and observers are in large part due to practitioners overestimating their levels of adherence and competence, and has been shown in both manualized research treatments and routine practices (Hogue et al., 2015). For example, in a summary of findings from trials with a comparatively large number of recorded sessions of addiction practitioners delivering standard treatment (e.g., CBT and the 12 Steps program), Carroll et al. (2010) found that the practitioners frequently overestimated their time spent on EBT. Moreover, EBT components occurred at low levels and in less than five percent of all sessions. Instead, clinician-initiated unrelated discourse (i.e., *chat*) was one of the more frequently observed interventions.

Motivational interviewing (MI) is a collaborative, client-centered and directional method for strengthening clients’ motivation to change (Miller and Rollnick, 2013), implemented in a variety of healthcare settings (Miller and Moyers, 2017). However, results from previous MI training research shows that also MI practitioner often overestimates their levels of fidelity compared to objective observers (Miller and Mount, 2001; Miller et al., 2003, 2004; Martino et al., 2009; Decker et al., 2013; Wain et al., 2015). The most used instrument for measuring MI fidelity is the Motivational Interviewing Treatment Integrity code (MITI) (Moyers et al., 2016). MITI 3.1 comprises two parts: (1) The five global ratings (Empathy, Evocation, Collaboration, Autonomy, and Direction), which provide an overall assessment of the practitioner’s performance on a five-point scale; and (2) The behavior counts, which are the frequency of the MI-practitioner’s utterances coded in seven different categories (Giving information, MI adherent behaviors, MI non-adherent behaviors, Closed questions, Open questions, Simple reflections, and Complex reflections). The coding instrument also includes recommended indicators of MITI proficiency to aid the evaluation of clinicians’ skillfulness in MI (Moyers et al., 2010).

Within education, metacognition is a well-known concept. Defined as the students’ thinking about their own learning process (Benassi et al., 2014), metacognition is a crucial factor in students’ ability to evaluate progress and decide on strategies for improvement (Cutting and Saks, 2012; Gooding et al., 2017). Also in this area of research, repeated studies have shown students’ ability to assess their own performance as limited (Rohrer and Pashler, 2010; Benassi et al., 2014). However, when repeatedly tested, students’ assessments improve (Rohrer and Pashler, 2010; Cutting and Saks, 2012; Benassi et al., 2014). Additionally, active-learning techniques have also shown to increase metacognition within education (Gooding et al., 2017). An active use of fidelity tools, such as the MITI, during supervision might thereby be an efficient way for practitioners to learn how to more accurately estimate their levels of adherence and competence following MI training.

The objective of this study was to expand previous research by examining the relationship between participants’ self-reported MI skills and objectively assessed MI skills over a period of 12 month that included six monthly supervision sessions. Two different types of supervision were included: Regular group supervision, or individual telephone supervision based on objective MITI feedback. In line with previous findings, we hypothesized that the relationships between participants’ self-reported skills and objectively assessed skills would be overall weak. However, we also hypothesized that the associations would increase over time, and that the associations would be stronger in the group that received monthly individual supervision sessions based on the MITI.

## Methods

Data were obtained from an MI implementation study (Beckman et al., 2021) conducted from September 2014 to January 2017 at the Swedish National Board of Institutional Care (SiS), a Swedish government agency for young people with psychosocial problems and adults with substance use disorders. The main aim of the study were to assess MI skills within the agency, and to evaluate two forms of MI supervision on the supervisor-supervisee working alliance, the supervisees’ feelings of discomfort/distress and MI skill acquisition. The analyses did not show any form of supervision as more effective, or that the MITI feedback did evoke negative emotions, or negatively affected the supervisor-supervisee working alliance or the supervisee’s skill acquisition (Beckman et al., 2021).

### Participants

Participants were 134 employees from 12 SiS institutions who previously had received at least one MI workshop during their employment at SiS. Demographic variables are presented in Table 1.

**TABLE 1:** Baseline characteristics of the enrolled participants.

Participants	SiS-GS (*n* = 64) *n* (%)	ITS (*n* = 70) *n* (%)	Total (*n* = 134) *n* (%)
**Gender**			
Male	23 (35.94)	27 (38.57)	50 (37.31)
Female	41 (64.06)	43 (61.43)	84 (62.67)
**Age**			
Mean age (*SD*)	42.52 (10.84)	43.79 (9.63)	43.16 (10.23)
**Education**			
College/higher degree	23 (35.94)	22 (31.43)	45 (33.58)
No higher degree	29 (45.31)	24 (34.29)	53 (39.55)
Missing	12 (18.75)	24 (34.29)	36 (26.87)
**Occupation**			
Assistant nurse	2 (3.13)	1 (1.43)	3 (2.24)
Head of institution	2 (3.13)	2 (2.86)	4 (2.99)
Nurse	1 (1.56)	2 (2.86)	3 (2.24)
Substance abuse counselor	46 (71.88)	43 (61.43)	89 (66.42)
Treatment program manager	0 (0.00)	2 (2.86)	2 (1.49)
Treatment administrator	6 (9.38)	8 (11.43)	14 (10.45)
Other	2 (3.13)	1 (1.43)	3 (2.24)
Missing	5 (7.81)	11 (15.71)	16 (11.94)

SiS, The Swedish National Board of Institutional Care; SiS-GS, regular group supervision; ITS, individual telephone supervision.

### Procedure

**FIGURE 1:**
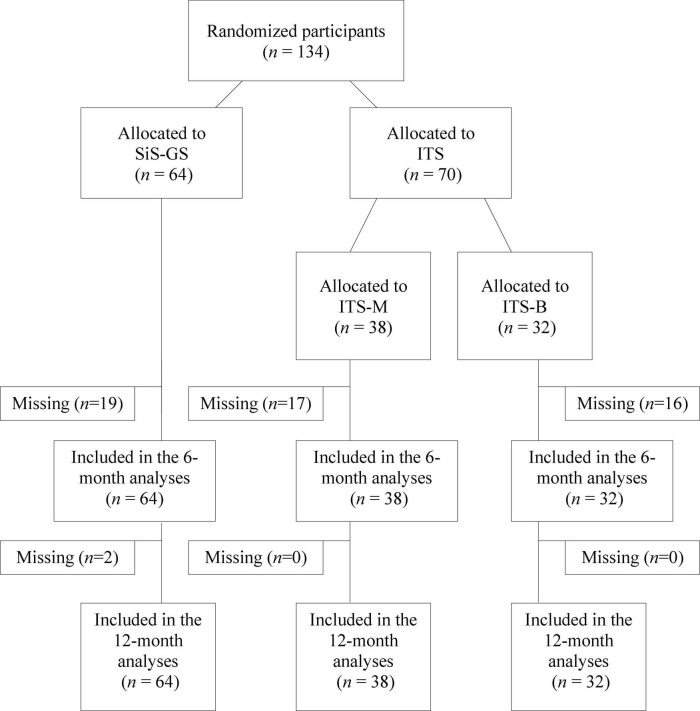
Flow diagram. SiS, The Swedish National Board of Institutional Care; SiS-GS, SiS group supervision; ITS, individual telephone supervision; ITS-B, individual telephone supervision including systematic feedback based on only the behavior counts part of the objective protocol; ITS-M, individual telephone supervision including systematic feedback based on the entire objective protocol.

All participants signed informed consent before participating in the study (the Regional Ethical Review Board in Stockholm, Sweden; dnr. 2013/904-31). They were then randomized to: (1) Six months of regular group supervision at their SiS-institutions (SiS-GS, *n* = 64), or (2) Six monthly individual telephone supervision sessions based on two types of MITI feedback (ITS, *n* = 70) (Figure 1). The randomization was conducted with a random number generator without stratification. Within SiS, a variety of substance abuse treatment options are available, including MI. In addition, all verbal interactions with clients must be performed in accordance with MI. SiS therefore offers all personnel who interact with clients a four- to five-day MI workshop with subsequent group supervision. This SiS regular group supervision is conducted at all institutions and is open to employees with at least one completed workshop in MI. The regular supervision session content varied slightly at the different SiS institutions during the course of the study (e.g., reports of/listening to self-selected parts of sessions from some of the participants, discussions, coaching, and role-plays). The monthly 30 min individual telephone supervision sessions were manual based and are described in more detail elsewhere (Beckman et al., 2017, 2019). All participants recorded three 20-min MI-sessions at their institution, either with a client or a real play (i.e., one practitioner recounts a personal experience to the other, who act as a therapist in relation to that situation) together with a colleague: At baseline, six (the 6-month assessment), and 12 (the 12-month follow-up) months after the baseline recording. The ITS group recorded four extra sessions in between baseline and the 6-month assessment, and received individual telephone supervision after all recordings except the 12-month follow-up. Following each recording, all participants self-assessed their MI skills in a questionnaire mirroring the MITI.

### Assessment

The recorded MI sessions were assessed for proficiency (i.e., adherence and competence) by coders at MIC Lab, now named the Motivational Interviewing Quality Assurance (MIQA) group at Karolinska Institutet (KI) in Stockholm, with a translated version of the MITI, version 3.1 (Forsberg et al., 2011a). All MIQA coders complete 120 h of training, take part in weekly group codings, and double-code 12 randomly selected recordings twice a year. At the middle of the study period, the ICC for the MITI variables ranged between good to excellent for all variables except for *Direction* and *MI non-adherent*, for which ICC was considered fair (Table 2). The Clinical Experience Questionnaire (CEQ) (Forsberg et al., 2011b) was used to self-assess MI skills. CEQ contains nine items: The first five correspond to each of the five global variables of the MITI (i.e., *Empathy, Evocation, Collaboration, Autonomy*, and *Direction*). In the following four, participants are asked to estimate the proportion of the behavior counts using a three-point scale (i.e., more *Reflections* than *Questions*, roughly the same, or more *Questions* than *Reflections*; more *Complex reflections* than *Simple reflections*, roughly the same, or more *Simple reflections* than *Complex reflections*; more *Open questions* than *Closed questions*, roughly the same, or more *Closed* than *Open questions*; more *MI adherent* utterances than *MI non-adherent* utterances, roughly the same, or more *MI non-adherent* than *MI adherent* utterances).

**TABLE 2:** The MIQA coder’s inter-rater reliability, assessed with a two-way mixed model with absolute agreement, single measures, ICC.

MITI variable	ICC	MITI variable	ICC
Empathy	0.69	MI adherent behaviors	0.82
Evocation	0.44	MI non-adherent behaviors	0.58
Collaboration	0.71	Closed questions	0.97
Autonomy	0.79	Open questions	0.98
Direction	0.44	Simple reflections	0.82
Giving information	0.62	Complex reflections	0.75

MITI, motivational interviewing treatment integrity code; ICC, intraclass correlation coefficient. According to Cicchetti’s (1994) system for evaluating intraclass correlations, an ICC below 0.40 is considered poor, an ICC between 0.40–0.59 is considered fair, an ICC between 0.60–0.74 is considered good, and an ICC between 0.75–1.00 is considered to be excellent (Cicchetti and Sparrow, 1981).

### Data analyses

The inter-rater agreement of the MITI coding was assessed with a two-way mixed model with absolute agreement, single measures, ICC. Due to non-normally distributed data, Spearman’s rho were used to test the correlations between the nine MITI proficiency measures and the nine CEQ-scores for each supervision group at baseline, the 6-month assessment and the 12-month follow-up. The Bonferroni correction was applied for multiple comparisons with the significance cut-off at *p* < 0.05. To examine whether the correlation coefficients were significantly different, the Fisher r-to-z transformation was used. To test the effectiveness of the different types of supervision on self-rating of competence, new variables based on the difference between the participants’ self-reported MI skills and the objectively assessed MI skills were created, and a generalized linear mixed model (GLMM) was then used to analyze both main (i.e., group, and time) and interaction (group X time) effects. Descriptive statistics, such as QQ-plots, showed which distribution best represented the data (i.e., nesting, repeated measures within individuals and random intercept for individuals). For one variable where we found a significant baseline difference, we included the baseline assessment as a fixed covariate to adjust for the initial imbalance. The Bonferroni correction was used for multiple comparisons within each GLMM analysis, and the intervention effect was calculated using Cohen’s d. All data were analyzed with the Statistical Package for the Social Sciences (SPSS), version 22.0.

## Results

Table 3 shows the correlations between the objectively assessed MITI proficiency measures and the participants’ self-reported CEQ-scores for the two groups at the three assessment points. Consistent with the first hypothesis, the associations between participants’ self-reports and the objective assessments were overall weak. Consistent with the second hypothesis, the discrepancy between self-reports and objective assessments (i.e., the MITI) then decreased over time: The GLMM-analysis showed significant time effects for four of the seven MITI proficiency measures: *Collaboration* [*F*(2, 113) = 4.97, *p* = 0.009, *d* = 0.42], *Reflection to questions* [*F*(2, 111) = 3.67, *p* = 0.029, *d* = 0.36], *Percent complex reflections* [*F*(2, 113) = 5.91, *p* = 0.004, *d* = 0.46], and *Percent MI-adherent* [*F*(2, 113) = 4.31, *p* = 0.016, *d* = 0.39].

**TABLE 3:** Correlations between the MITI proficiency measures and the practitioners’ self-assessment (i.e., the CEQ-scores) at the three assessment points.

	ITS			SiS-GS		
MITI variable	Baseline *n* = 57	6 month *n* = 41	12 month *n* = 35	Baseline *n* = 63	6 month *n* = 26	12 month *n* = 27

	*r*_*s*_ (*P*-value)	*r*_*s*_ (*P*-value)	*r*_*s*_ (*P*-value)	*r*_*s*_ (*P*-value)	*r*_*s*_ (*P*-value)	*r*_*s*_ (*P*-value)
Empathy	0.00 (0.990)	0.20 (0.216)	0.04 (0.830)	–0.01 (0.915)	–0.02 (0.917)	0.31 (0.115)
Evocation	–0.23 (0.102)	0.00 (0.991)	0.13 (0.457)	0.02 (0.905)	0.36 (0.087)	0.14 (0.488)
Collaboration	–0.30 (0.025)	0.11 (0.480)	–0.32 (0.065)	0.04 (0.781)	0.37 (0.066)	0.56 (0.003)***
Autonomy Support	–0.13 (0.330)	–0.04 (0.792)	–0.11 (0.521)	0.08 (0.541)	0.28 (0.074)	0.08 (0.690)
Direction	0.14 (0.341)	0.01 (0.934)	0.06 (0.727)	0.26 (0.061)	0.23 (0.293)	0.44 (0.024)
Reflection to questions	0.22 (0.108)	0.00 (0.990)	0.24 (0.164)	0.34 (0.006)	0.37 (0.064)	0.70 (< 0.001)*
% Complex reflections	0.13 (0.320)	–0.02 (0.882)	–0.14 (0.416)	0.09 (0.470)	0.53 (0.006)*	0.09 (0.663)
% Open questions	–0.06 (0.683)	0.28 (0.074)	0.29 (0.097)	0.02 (0.069)	0.35 (0.083)	0.35 (0.071)
% MI-adherent	–0.03 (0.830)	0.12 (0.455)	–0.02 (0.899)	0.33 (0.009)*	0.05 (0.823)	0.16 (0.420)

MITI, the Motivational Interviewing Treatment Integrity Code; CEQ, the Clinical Experience Questionnaire; ITS, the individual telephone supervision group; SiS-GS, the SiS group supervision group; r_s_, Spearman correlation coefficient. */***The two groups correlations differed significantly at the 0.05 (*) or at the 0.001 (***) level at this time point on these MITI proficiency measures.

Although the correlations were persistently higher for the SiS-GS group, only one out of nine was significantly higher at the 6-month assessment (i.e., *Percent complex reflections*), and only two were significantly higher at the 12-month follow up (i.e., *Collaboration* and *Reflection to question*) (Table 3). Additionally, contrary to the third hypothesis, the two groups did not develop differently over time: The GLMM-analysis showed no significant interaction or group effects.

## Discussion

This study examined the relationship between practitioners self-reported MI skills and objectively assessed MI skills. The participants received either 6 months of regular institutional group supervision, or six monthly individual telephone supervision sessions based on objective feedback. The associations between self-reported and objectively assessed MI skills were overall weak, but both groups’ weak correlations then increased somewhat over time. However, the associations between self-reported and objectively assessed MI skills were not stronger in the group that received individual telephone supervision based on objective feedback.

Practitioners’ difficulties in assessing their own performance have repeatedly been shown in previous studies (Carroll et al., 1998, 2010; Brosan et al., 2008; Beidas and Kendall, 2010). However, within educational research, metacognition is well-known and numerous studies have shown that estimates become more accurate during recurrent testing (Rohrer and Pashler, 2010; Cutting and Saks, 2012; Benassi et al., 2014). In this study, the ITS group’s MI skills were tested in six monthly recordings followed by supervision based on the results of objective assessment of those recordings (i.e., the MITI). Though participants’ self-reported MI skills were never a part of these supervision sessions. The MITI results were a regular part of every session, but they were never compared with the participants’ self-reports. In fact, none of the participants got the chance to see their self-reports once they were handed in. The SiS-GS group was also tested during the course of the study, but only three times as opposed to seven for the ITS group. Thus, six monthly recordings and subsequent supervision sessions, including detailed compilations of the objectively assessed skills, do not seem sufficient for practitioners to develop better estimates of their actual MI performances. On the contrary – for the group that received supervision as usual (i.e., SiS-GS) we found generally higher correlations between objective and subjective ratings. Notably, only 3 of these 18 sets of correlations were significantly higher at the 6-, and 12-month assessments (Table 3), and the two groups did not develop differently over time according to the GLMM analysis (no significant interaction). Nevertheless, the participants’ ability to self-assess increased somewhat in both groups over time (a Time-effect in the GLMM). The implementation study (Beckman et al., 2021) showed increased adherence and competence for the participants over time, and there may be a relationship between increased skills and the ability to self-assess.

Since previous research has highlighted metacognition as critical to learning and performance, and increasingly important as learners advance (Benassi et al., 2014), an additional way to use fidelity tools for enhanced learning during supervision might be to actively work with the supervisee’s abilities to self-assess to thereby promote metacognition. Fidelity based self-assessment as a supervision tool may also inform practitioners on opportunities for development, and thereby contribute to a more efficient training (Cutting and Saks, 2012). By comparing self-rated assessment with independent observers during supervision sessions, practitioners’ ability to self-assess may also be improved. Since evaluation of practitioners’ competence and adherence relies heavily on self-reports, low ability in this domain has serious implications for both effective supervision and practice. Discrepancy in perception and actual behavior is especially problematic when practitioners are overestimating their levels of adherence and competence, given the findings of better outcomes when treatments is conducted with fidelity (Beidas and Kendall, 2010).

This study has several limitations: The implementation study had recruitment difficulties, the participants were self-selected, and 40.3 percent of the participants dropped out, which limits the generalizability of the findings. Additionally, the coders were not blind to the group allocation, and the same coders both rated the sessions and conducted the supervision. This procedure may have affected the coding reliability (Moyers et al., 2016). Despite these limitations, the present study contributes to the knowledge of the relationship between self-reported and objectively assessed MI skills by being one of few studies that compare the accuracy of practitioners’ self-assessment in two different supervision groups using a relatively large sample in a naturalistic setting over a period of 12 month, and can therefore provide some direction for future MI training and implementation studies.

### Conclusion

This study contributes to the field by confirming and expanding previous findings on the inaccuracy of practitioners’ self-reports. The ability to self-assess skills after MI-training seems to increase somewhat over time, but six monthly recordings and subsequent MI supervision sessions, including detailed compilations of the objectively assessed skills, were not sufficient for the SiS-practitioners in this study to develop better estimates of actual performances. An additional way to use fidelity tools, such as the MITI, for enhanced learning during supervision could be to promote metacognition. Fidelity based self-assessment as a supervision tool may also inform practitioners on opportunities for development and thereby contribute to a more efficient training, and might also enhance practitioners’ ability to self-assess.

### Practice Implications

The results shed a light on practitioners’ difficulties in assessing their own competence. Practitioners’ ability to self-assess may increase with specific training, but to ensure that patients receive intended care, adherence and competence should in any case be assessed objectively.

## Data availability statement

The raw data supporting the conclusions of this article will be made available by the authors, without undue reservation.

## Ethics statement

The studies involving human participants were reviewed and approved by the Regional Ethical Review Board in Stockholm, Sweden; dnr. 2013/904-31. The patients/participants provided their written informed consent to participate in this study.

## Author contributions

MB: conceptualization, methodology, data curation, formal analysis, funding acquisition, and writing – original draft, review and editing. HL: conceptualization, methodology, formal analysis, and writing – review and editing. LÖ: project administration and writing – review and editing. LF: conceptualization, methodology, funding acquisition, and writing – review and editing. TL: resources, supervision and writing – review and editing. AG: conceptualization, methodology, data curation, formal analysis, supervision, and writing – review and editing. All authors contributed to the article and approved the submitted version.

## Conflict of interest

The authors declare that the research was conducted in the absence of any commercial or financial relationships that could be construed as a potential conflict of interest.

## Publisher’s note

All claims expressed in this article are solely those of the authors and do not necessarily represent those of their affiliated organizations, or those of the publisher, the editors and the reviewers. Any product that may be evaluated in this article, or claim that may be made by its manufacturer, is not guaranteed or endorsed by the publisher.
